# Study on the clinical efficacy of repetitive transcranial magnetic stimulation in adolescent patients with depression and its influence on neurotransmitters based on functional near-infrared spectroscopy

**DOI:** 10.3389/fpsyt.2025.1698181

**Published:** 2025-12-04

**Authors:** Li Zhang, Xia Lan, Juan Chen, Jiacheng Long, Xinmei Xiang, Lin Wang, Rongfang He

**Affiliations:** 1 Department of Dermatology, The Affiliated Hospital of Southwest Medical University, Luzhou, Sichuan, China; 2 Nursing Department of Luzhou Mental Health Center, Luzhou, Sichuan, China; 3 Department of Psychiatry, The Affiliated Hospital of Southwest Medical University, Luzhou, Sichuan, China; 4 Fundamental and Clinical Research on Mental Disorders Key Laboratory of Luzhou, Luzhou, Sichuan, China; 5 School of Nursing, Southwest Medical University, Luzhou, Sichuan, China; 6 Department of Nursing, The Affiliated Hospital of Southwest Medical University, Luzhou, Sichuan, China

**Keywords:** fNIRS, repetitive transcranial magnetic stimulation, adolescents, depression, neurotransmitters

## Abstract

**Objective:**

This study aims to investigate the clinical efficacy of rTMS on adolescent patients with depression and its impact on neurotransmitter levels using fNIRS technology.

**Methods:**

Eighty adolescents with major depressive disorder who attended the Psychosomatic Medicine Outpatient Clinic of the Affiliated Hospital of Southwest Medical University between August 2024 and January 2025 were enrolled and randomly assigned to control (n = 40; sertraline hydrochloride + sham rTMS) or research (n = 40; sertraline hydrochloride + active rTMS) groups. All participants received 4 weeks of treatment. Depression severity (HAMD), anxiety severity (HAMA), quality of life (GQOLI-74), cognitive performance (DSST, DST, SVF), fNIRS-derived cortical metrics (frontal and bilateral temporal integral and centroid values), and serum neurotransmitter levels (DA, 5-HT, NE) were assessed at baseline, week 2, and week 4.

**Results:**

Baseline characteristics were comparable (*P* > 0.05). Two-factor repeated measures analysis of variance indicated that for the scores of each dimension of HAMD, HAMA, GQOLI-74 and the levels of neurotransmitters, the interaction and main effects of time and group were all significant (all *P* < 0.05). Simple effect analysis revealed that after the intervention, the above scores and neurotransmitter levels of the research group improved more than those of the control group, and the levels within the group also significantly improved compared to the baseline levels (all *P* < 0.05). In terms of cognitive function and most fNIRS indicators, although the interaction was not significant, the scores of the research group after the intervention and the integral values of the frontal lobe and bilateral temporal lobes were significantly higher than those of the control group, and both groups significantly improved compared to the baseline (all *P* < 0.05). In addition, the interaction of the bilateral temporal lobe center of gravity was significant, and the decrease in the study group after the intervention was greater than that of the control group (*P* < 0.05).

**Conclusion:**

rTMS significantly alleviates depression and anxiety, enhances quality of life and cognitive performance, boosts frontotemporal activity, and increases monoamine neurotransmitter levels in adolescents with major depressive disorder, warranting wider clinical adoption.

## Introduction

Depression, a psychiatric disorder characterized primarily by low mood and loss of interest, is increasingly prevalent among adolescents, now ranking as one of the most significant mental health issues in this age group (1). Compared to adults, the etiology of depression in adolescents is more complex, involving a combination of immature neurodevelopment, dramatic hormonal changes, and psychosocial stressors, which not only impair their emotional and cognitive functions but also may lead to long-term social dysfunction and an increased risk of suicide (2). Therefore, effective interventions for this particular group are crucial.

Currently, the first-line treatment options for adolescent depression primarily rely on medications such as selective serotonin reuptake inhibitors (SSRIs) and psychotherapy. However, pharmacological treatments often come with adverse effects like nausea, insomnia, and weight changes, leading to poor compliance or suboptimal efficacy in some patients (3); psychotherapy is limited by long treatment durations and a scarcity of therapist resources (4). These limitations have prompted the exploration of safer, more efficient, and better-tolerated novel therapeutic approaches. Repetitive transcranial magnetic stimulation (rTMS), as a non-invasive brain stimulation technique, has demonstrated significant efficacy in adult depression, particularly in treatment-resistant cases, by modulating neural activity in the frontal cortex, which is closely related to mood regulation (5). Compared to traditional medications, rTMS offers potential advantages such as fewer side effects, non-invasiveness, and strong targeting, providing a promising treatment option for adolescent patients (6).

Nevertheless, the application of rTMS in adolescents requires careful evaluation. Given that their brains are still in a critical period of development, the mechanism of action of rTMS and its long-term effects on the nervous system may differ from those in adults. Previous studies have largely focused on the improvement of clinical symptoms, with insufficient exploration of the underlying neurobiological mechanisms, which limits a deeper understanding and optimization of the efficacy of rTMS. Functional near-infrared spectroscopy (fNIRS) technology enables non-invasive, real-time monitoring of cerebral cortical hemodynamic activity, providing a unique window for indirectly observing neural activity and metabolic status (7). By utilizing fNIRS, it is expected to directly reveal how rTMS modulates the function of key brain regions in adolescents with depression and to bridge the connection between these modulations and changes in neurotransmitters. Current theories suggest that rTMS may exert therapeutic effects by regulating monoamine neurotransmitters such as dopamine (DA) and serotonin (5-HT) (8), but this hypothesis still lacks direct clinical evidence in the adolescent population.

Therefore, to clarify the clinical value of rTMS for adolescent depression and to elucidate its mechanism of action, this study aims to combine fNIRS with neurotransmitter detection to systematically evaluate the efficacy of rTMS and to explore in depth its impact on frontal and temporal lobe brain function and key neurotransmitter levels. This is expected to provide a solid theoretical and empirical basis for the precise application of this therapy.

## Materials and methods

### Subjects

From August 2024 to January 2025, 80 adolescent patients with depression were recruited from the outpatient department of Psychosomatic Medicine at the Affiliated Hospital of Southwest Medical University for this study. They were randomly assigned to a control group (conventional sertraline hydrochloride combined with sham rTMS treatment) and a research group (conventional sertraline hydrochloride combined with active rTMS treatment), with 40 cases in each group. The control group received conventional sertraline hydrochloride combined with sham rTMS treatment, while the research group received conventional sertraline hydrochloride combined with active rTMS treatment. During the study, no subjects dropped out due to loss of follow-up or side effects, and 40 data were finally completed in both groups. Both patients and their families signed informed consent forms. This study was approved by the Ethics Committee of Southwest Medical University (approval number: 20240723).

Inclusion Criteria: Age between 10 and 19 years; diagnosis of depressive episode or recurrent depressive disorder according to the International Statistical Classification of Diseases and Related Health Problems, Tenth Revision (ICD-10) (9) confirmed by two psychiatrists with the title of attending physician or above; a total score of ≥17 points on the 17-item Hamilton Depression Rating Scale (HAMD-17) at screening and baseline; right-handed and not color-blind; first-time recipients of relevant treatment and no use of antipsychotic medications within the past month.

Exclusion Criteria: Failure of vital organ function; presence of severe suicidal ideation or complete refusal of treatment; comorbid psychotic disorders or presence of psychotic symptoms, substance dependence or abuse, or intellectual disability; history of epilepsy, brain disorders, or other severe somatic diseases; inability to take medication voluntarily or poor medication adherence; abnormal electroencephalogram (EEG) results or presence of metallic implants in the body; significantly elevated intracranial pressure.

### Treatment protocol

Control Group: The control group received conventional treatment with sertraline hydrochloride combined with sham repetitive rTMS treatment. rTMS was administered 5 times per week, with each session lasting 20 minutes, for a total duration of 4 weeks.

research group: The research group received conventional treatment with sertraline hydrochloride combined with active rTMS treatment. rTMS was administered 5 times per week, with each session lasting 20 minutes, for a total duration of 4 weeks.

rTMS Treatment Protocol: The rTMS treatment was conducted using the CCY-IIIA type repetitive transcranial magnetic stimulator produced by Wuhan Yirede Company. During treatment, participants were positioned in a comfortable posture and instructed to relax. The target stimulation sites were the left and right dorsolateral prefrontal cortex (DLPFC). The localization method for DLPFC was as follows: first, the Cz point was determined according to the international 10–20 EEG system, and then moving 6 cm forward from the Cz point would reach the DLPFC area. The precise localization and recording were performed using a neuronavigation system or scalp marking method. For the research group, the center of the “figure-eight” coil was placed over the left and right DLPFC, with the coil in close contact with the scalp. The stimulation intensity was set at 100% of the motor threshold (MT); the stimulation frequency was 1 Hz, with 1200 pulses per day on the left side and 600 pulses per day on the right side. Each treatment session lasted 20 minutes, conducted from Monday to Friday, for a total of 4 consecutive weeks, accumulating to 20 sessions. For the control group, all procedures were identical to those of the research group, except that no effective magnetic field was provided.

### Observation index

The primary outcome measures were the Hamilton Depression Scale (HAMD) and the Hamilton Anxiety Scale (HAMA), while secondary outcome measures included Quality of Life, Cognitive Function, fNIRS, and Neurotransmitter Levels. Data were collected at three time points: before treatment, at the end of the second week of treatment, and at the end of the treatment. The mid-term assessment was designed to dynamically monitor patients’ treatment responses and safety. It also helped to precisely delineate the temporal trajectory of the clinical efficacy of rTMS, thereby identifying its critical time window of effectiveness.

Baseline Characteristics: Data were collected on gender, age, BMI, course of disease, educational level, and place of residence.

Depressive Symptoms: The Hamilton Depression Scale (HAMD) (10) was used to assess depressive symptoms before intervention, two weeks after intervention, and at the end of the intervention. The 24-item version of the HAMD was employed in this study. A total score of less than 8 indicates the absence of depressive symptoms; a score of ≥20 suggests mild to moderate depression; and a score of ≥35 indicates severe depression.

Anxiety Symptoms: The Hamilton Anxiety Scale (HAMA) (11) was used to assess anxiety symptoms before intervention, two weeks after intervention, and at the end of the intervention. Scores below 7 indicate no anxiety symptoms; 7–14 points indicate mild anxiety; 15–21 points indicate moderate anxiety; 22–29 points indicate marked anxiety; and scores above 29 indicate severe anxiety.

Quality of Life: The Generic Quality of Life Inventory-74 (GQOLI-74) (12) was used to evaluate the quality of life before the intervention, two weeks after the intervention, and at the end of the intervention. The assessment included physical functioning, psychological functioning, social functioning, and material well-being. Higher scores indicate better quality of life.

Cognitive Function: Cognitive function was assessed using the Digit Symbol Substitution Test (DSST), Digit Span Test (DST), and Semantic Verbal Fluency Task (SVF) before the intervention, two weeks after the intervention, and at the end of the intervention. DSST: This test evaluates the participants’ attention and psychomotor speed. Participants were provided with a key matching digits (0–9) to corresponding symbols. After practicing with 10 digits, they were required to fill in the symbols corresponding to each digit as quickly and accurately as possible within 90 seconds. One point was awarded for each correctly filled symbol, with zero points for incorrect or unfilled symbols. The maximum score was 90 points. DST: This test assesses immediate memory, attention, and working memory. It consists of forward and backward tests. The evaluator read aloud a sequence of digits at a uniform pace. For the forward test, participants were asked to repeat the digits in the same order, while for the backward test, they were required to repeat the digits in reverse order. The forward test included sequences ranging from 3 to 12 digits, and the backward test included sequences ranging from 2 to 10 digits. Testing began with the shortest sequence length, with two attempts allowed for each sequence length. If participants succeeded on the first attempt, they proceeded to the next sequence length; if they failed on the first attempt, they were given a second attempt. The test was discontinued if both attempts failed. The maximum score for the forward test was 12 points, and for the backward test, it was 10 points, with a total possible score of 22 points. SVF: This test evaluates verbal function. Participants were asked to name as many words as possible within a specified category (in this study, the category was “animals”) within 60 seconds. One point was awarded for each correctly named animal. Repeated words, words not fitting the animal category, or fabricated words were not scored.

fNIRS Brain Function Imaging Indicators: fNIRS brain function imaging indicators were assessed before the intervention, two weeks after the intervention, and at the end of the intervention using a multi-channel fNIRS system (NIRSport, NIRx Medical Technologies, LLC). This system measures hemodynamic neuroactivation via changes in oxy-, deoxy-, and total hemoglobin in the cerebral cortex. The probes were arranged according to the international 10–20 EEG system, with 16 light source emitters and 15 detectors alternately placed to form 40 measurement channels. The central optical probe was positioned at FPz, with the lowest probes aligned along the Fp1-Fp2 line. The light sources and detectors were symmetrically placed over the prefrontal cortex (PFC), sensorimotor cortex (SMC), and premotor and supplementary motor cortex (PMC/SMA) regions on both sides. Each participant’s fNIRS hemodynamic characteristics were recorded, including the integrated values and centroid values of the PFC, as well as the integrated and centroid values of the bilateral temporal lobes. The measurements were conducted in a quiet, enclosed environment. Participants wore the fNIRS cap and sat upright in a chair with their eyes closed, relaxing their thoughts and minimizing movement for at least 6 minutes of resting-state data collection.

Neurotransmitter Levels: Blood samples (4 mL) were collected by venous blood sampling before the intervention, two weeks after the intervention, and at the end of the intervention. The samples were centrifuged at 3500 rpm for 12 minutes. Levels of DA, 5-HT, and norepinephrine (NE) were measured using enzyme-linked immunosorbent assay (ELISA).

### Statistical method

Statistical analysis and graphs were performed with SPSS 17.0 and GraphPad Prism 8.0. Quantitative data are presented as mean ± SD. Normality was verified by Shapiro–Wilk test and homogeneity of variances by Levene’s test (*P* > 0.05). A two-way (group × time) repeated-measures ANOVA was used to examine interaction and main effects. When the interaction was significant, simple-effects analyses were conducted: independent-samples t-tests were applied between groups at each time point, and paired t-tests were used within each group across time points. All *post-hoc* comparisons were Bonferroni-adjusted, with the significance level set at α = 0.05/number of comparisons. When the interaction was not significant but a main effect was, only the main effect is reported. Categorical data are expressed as n (%) and compared between groups with the *χ*² test or Fisher’s exact test when expected frequencies were < 5. All tests were two-tailed; *P* < 0.05 was considered statistically significant.

## Results

### Baseline data

There was no significant difference in baseline data between the two groups (*P* > 0.05), which was comparable (**Table 1**).

**Table 1:** Comparison of baseline data between the two groups [(n, %), mean ± SD].

Indicators		Control group (n = 40)	Research group (n = 40)	*t/χ*²	*P* value
Gender	Male	12 (30.00)	10 (25.00)	0.251	0.617
	Female	28 (70.00)	30 (75.00)		
Age, years		15.33 ± 1.46	14.93 ± 1.85	1.074	0.286
BMI, kg/m^2^		20.06 ± 2.19	19.43 ± 1.87	1.384	0.170
Course of disease, years		2.27 ± 0.45	2.36 ± 0.54	0.810	0.421
Educational level	Junior high school and below	10 (25.00)	14 (35.00)	0.952	0.329
	high school	30 (75.00)	26 (65.00)		
Place of residence	Towns	26 (65.00)	31 (77.50)	1.526	0.217
	Village	14 (35.00)	9 (22.50)		

BMI, Body Mass Index.

### Depressive status

**Figure 1:**
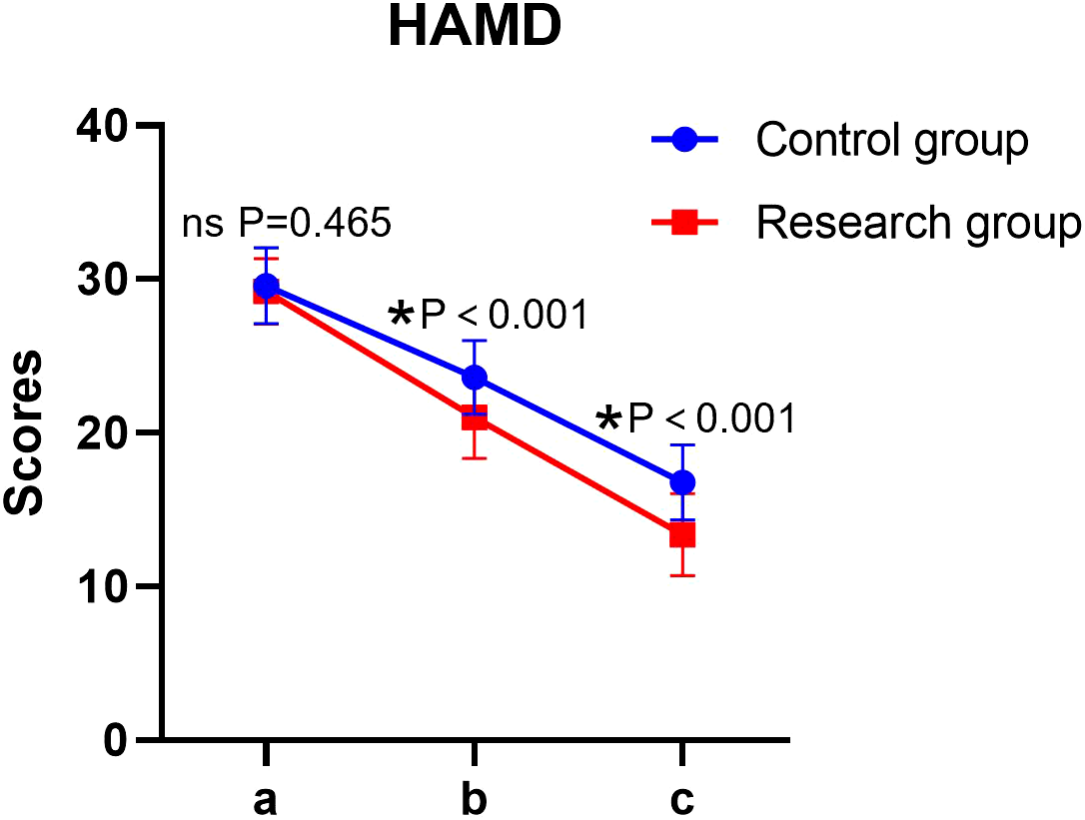
HAMD scores in both groups. a: before the intervention, b: two weeks after the intervention, and c: at the end of the intervention. HAMD, Hamilton Depression Rating Scale. ns, no significance, *P < 0.05.

Significant main effects of time and group, as well as a significant time × group interaction, were observed for HAMD scores (all *P* < 0.05). The simple effect analysis indicated that there was no statistically significant difference in HAMD scores between the two groups before the intervention (*P* > 0.05); at the end of the intervention, the score of the research group was lower than that of the control group, and the difference was statistically significant (*P* < 0.05). The intra-group comparison showed that the HAMD scores of both groups decreased significantly after the intervention compared to the baseline (*P* < 0.05) (**Table 2**, **Figure 1**).

**Table 2:** HAMD scores in both groups (n=40, mean ± SD).

Group	Control group	Research group	*F* (*P*)
Before the intervention	29.58±2.47	29.20±2.15	*F*_time_ (*P*): 667.241 (< 0.001)
Two weeks after the intervention	23.63±2.40^*^	21.03±2.67^*^	*F*_group_ (*P*): 44.123 (< 0.001)
At the end of the intervention.	16.78±2.45^*#^	13.38±2.68^*#^	*F*_Interaction_ (*P*): 8.002 (< 0.001

Compared with before intervention, ^*^P < 0.05; Compared with the same group after two weeks of intervention, ^#^*P* < 0.05.

### Anxiety status

**Figure 2:**
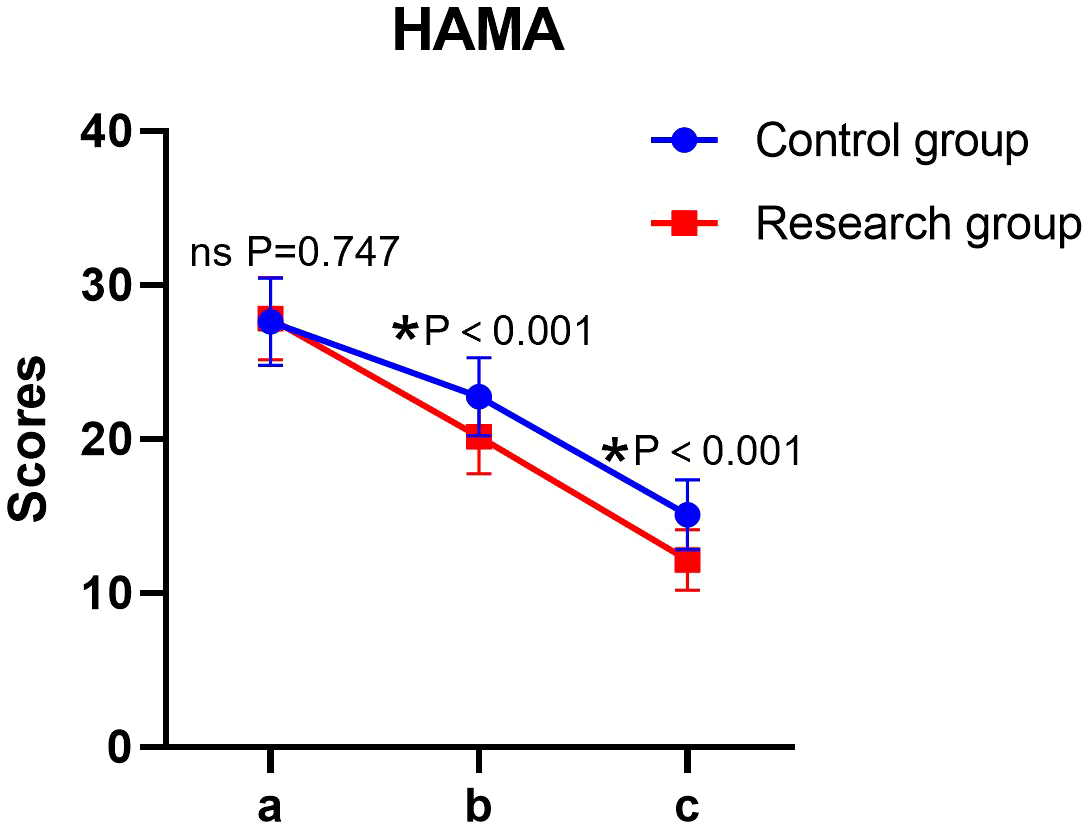
HAMA scores of patients in both groups. a: before the intervention, b: two weeks after the intervention, and c: at the end of the intervention. HAMA, Hamilton Depression Rating Scale. ns, no significance, *P < 0.05.

Significant main effects of time and group, as well as a significant time × group interaction, were found for HAMA scores (all P < 0.05). The simple effect analysis indicated that there was no statistically significant difference in HAMA scores between the two groups before the intervention (*P* > 0.05); at the end of the intervention, the score of the research group was lower than that of the control group, and the difference was statistically significant (*P* < 0.05). The intra-group comparison showed that the HAMA scores of both groups decreased significantly after the intervention compared to the baseline (*P* < 0.05) (**Table 3**, **Figure 2**).

**Table 3:** HAMA scores in both groups (n=40, mean ± SD).

Group	Control group	Research group	*F* (*P*)
Before the intervention	27.61±2.85	27.81±2.66	*F*_time_ (*P*): 653.555 (< 0.001)
Two weeks after the intervention	22.74±2.56^*^	20.16±2.40^*^	*F*_group_ (*P*): 30.709 (< 0.001)
At the end of the intervention.	15.09±2.29^*#^	12.15±1.98^*#^	*F*_Interaction_ (*P*): 9.614 (< 0.001)

Compared with before intervention, ^*^P < 0.05; Compared with the same group after two weeks of intervention, ^#^*P* < 0.05.

### Quality of life

**Figure 3:**
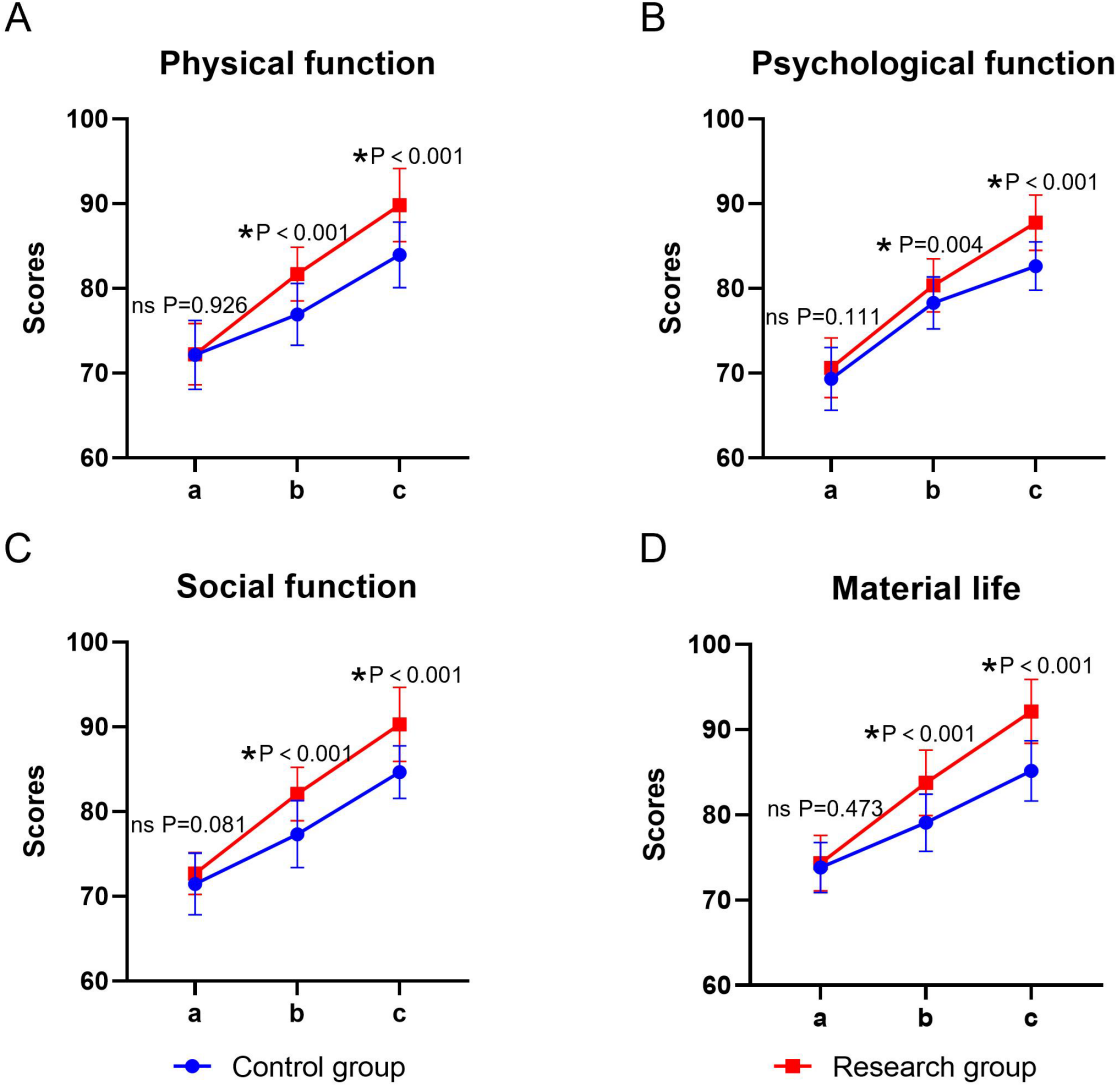
Quality of life in the two groups. **(A)** physical functioning, **(B)** psychological functioning, **(C)** social functioning, **(D)** material life; a: before the intervention, b: two weeks after the intervention, and c: at the end of the intervention. ns, no significance, *P < 0.05.

The main effect of time and group on the scores of each dimension of GQOLI-74 was significant, and the interaction between time × group was also significant (*P* < 0.05). The simple effect analysis indicated that before the intervention, there was no statistically significant difference in the scores of each dimension of GQOLI-74 between the two groups (*P* > 0.05); at the end of the intervention, the scores of GQOLI-74 in the research group were higher than those in the control group, and the difference was statistically significant (*P* < 0.05). The intra-group comparison showed that the scores of both groups significantly increased compared to the baseline level after the intervention (*P* < 0.05) (**Table 4**, **Figure 3**).

**Table 4:** Quality of life scores in both groups (n = 40, mean ± SD).

Dimension	Group	Control group	Research group	*F* (*P*)
Physical function	Before the intervention	72.15±4.06	72.23±3.63	*F*_time_ (*P*): 299.191 (< 0.001)
	Two weeks after the intervention	76.93±3.66^*^	81.68±3.16^*^	*F*_group_ (*P*): 52.821(< 0.001)
	At the end of the intervention.	83.95±3.87^*#^	89.83±4.32^*#^	*F*_Interaction_ (*P*): 13.094 (< 0.001)
Psychological function	Before the intervention	69.33±3.70	70.63±3.51	*F*_time_ (*P*): 441.707 (< 0.001)
	Two weeks after the intervention	78.28±3.06^*^	80.35±3.12^*^	*F*_group_ (*P*): 44.989 (< 0.001)
	At the end of the intervention.	82.63±2.86^*#^	87.74±3.26^*#^	*F*_Interaction_ (*P*): 7.576 (< 0.001)
Social function	Before the intervention	71.45±3.64	72.68±2.48	*F*_time_ (*P*): 386.292 (< 0.001)
	Two weeks after the intervention	77.33±3.96^*^	82.08±3.15^*^	*F*_group_ (*P*): 73.057 (< 0.001)
	At the end of the intervention.	84.65±3.11^*#^	90.28±4.36^*#^	*F*_Interaction_ (*P*): 8.837 (< 0.001)
Material life	Before the intervention	73.83±2.9	74.33±3.26	*F*_time_ (*P*): 353.665 (< 0.001)
	Two weeks after the intervention	79.08±3.35^*^	83.75±3.84^*^	*F*_group_ (*P*): 82.062 (< 0.001)
	At the end of the intervention.	85.15±3.54^*#^	92.13±3.76^*#^	*F*_Interaction_ (*P*): 17.968 (< 0.001)

Compared with before intervention, ^*^P < 0.05; Compared with the same group after two weeks of intervention, ^#^*P* < 0.05.

### Cognitive function

**Figure 4:**
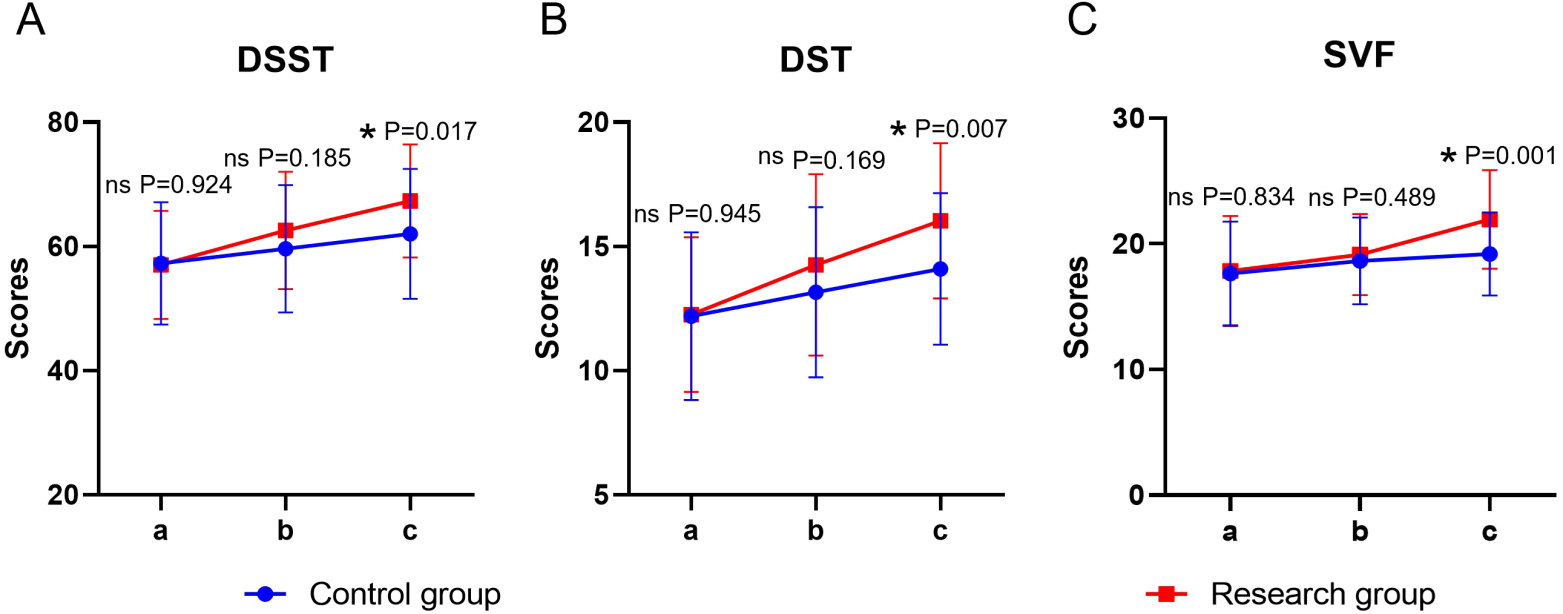
Cognitive function in both groups. **(A)** DSST, **(B)** DST, **(C)** SVF; a: before the intervention, b: two weeks after the intervention, a, c: at the end of the intervention. DSST, Digit Symbol Substitution Test; DST, Digit Span Test; SVF, Semantic Verbal Fluency Task. ns, no significance, *P < 0.05.

Both time and group showed significant main effects on cognitive function (*P* < 0.05), whereas the time × group interaction was not significant (*P* > 0.05). Before the intervention, there was no statistically significant difference in the DSST, DST, and SVF scores between the two groups (*P* > 0.05). At the end of the intervention, the scores of the above items in both groups were significantly higher than their respective baselines (*P* < 0.05), and the scores of the study group were significantly higher than those of the control group (*P* < 0.05) (**Table 5**, **Figure 4**).

**Table 5:** Cognitive function in both groups (n = 40, mean ± SD).

Group	Group	Control group	Research group	*F* (*P*)
DSST	Before the intervention	57.25±9.88	57.05±8.72	*F*_time_ (*P*): 12.104 (< 0.001)
	Two weeks after the intervention	59.63±10.27	62.58±9.43^*^	*F*_group_ (*P*): 4.657 (0.032)
	At the end of the intervention.	62.00±10.45^*^	67.33±9.10^*#^	*F*_Interaction_ (*P*): 1.646 (0.195)
DST	Before the intervention	12.20±3.38	12.25±3.12	*F*_time_ (*P*): 15.497 (< 0.001)
	Two weeks after the intervention	13.15±3.43	14.25±3.66^*^	*F*_group_ (*P*): 6.270 (0.013)
	At the end of the intervention.	14.10±3.06^*^	16.03±3.13^*#^	*F*_Interaction_ (*P*): 1.839 (0.161)
SVF	Before the intervention	17.63±4.12	17.83±4.38	*F*_time_ (*P*): 11.911 (< 0.001)
	Two weeks after the intervention	18.63±3.45	19.15±3.24	*F*_group_ (*P*): 5.926 (0.016)
	At the end of the intervention.	19.20±3.31	21.93±3.92^*#^	*F*_Interaction_ (*P*): 2.560 (0.079)

Compared with before intervention, ^*^P < 0.05; Compared with the same group after two weeks of intervention, ^#^*P* < 0.05.

### fNIRS brain functional imaging indicators

**Figure 5:**
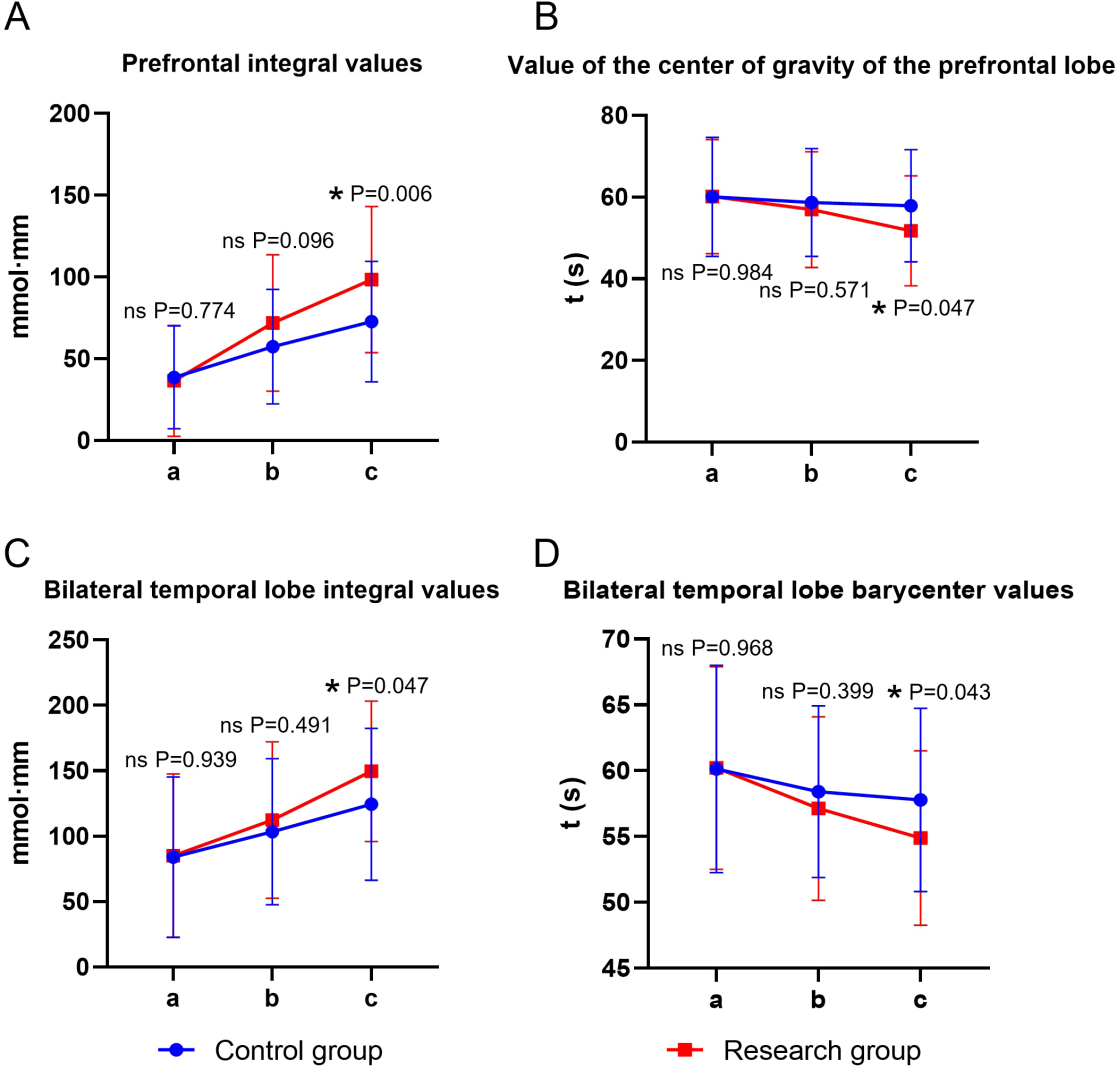
Functional brain imaging indices of fNIRS in both groups. **(A)** prefrontal integral value, **(B)** prefrontal center of gravity value, **(C)** bilateral temporal integral value, **(D)** bilateral temporal center of gravity value; a: before the intervention, b: two weeks after the intervention, and c: at the end of the intervention. ns, no significance, *P < 0.05.

Time and group showed significant main effects on prefrontal integral values, prefrontal gravity values, and bilateral temporal integral values (all *P* < 0.05), whereas the time × group interaction was not significant for any of these three outcomes (*P* > 0.05). For bilateral temporal barycenter values, both main effects and the time × group interaction were significant (*P* < 0.05). Before the intervention, there was no statistically significant difference in the fNIRS brain functional imaging indicators between the two groups (*P* > 0.05). At the end of the intervention, the integral values of the frontal lobe and bilateral temporal lobe in both groups were significantly higher than the baseline, and the center of gravity values were significantly lower (time main effect, *P* < 0.05). For the three indicators with insignificant interaction, the integral values of the study group after intervention were significantly higher than those of the control group, and the center of gravity values were significantly lower than those of the control group (group main effect, *P* < 0.05). For the bilateral temporal lobe center of gravity value (with significant interaction), the simple effect analysis showed that the decrease amplitude of the experimental group after intervention was significantly greater than that of the control group (*P* < 0.05) (**Table 6**, **Figure 5**).

**Table 6:** Functional brain imaging indices of fNIRS in both groups (n = 40, mean ± SD).

Group	Group	Control group	Research group	*F* (*P*)
Prefrontal integral values	Before the intervention	38.64±31.47	36.53±33.93	*F*_time_ (*P*): 31.421 (<0.001)
	Two weeks after the intervention	57.43±35.01*	71.91±41.63	*F*_group_ (*P*): 6.161 (0.014)
	At the end of the intervention.	72.69±36.83^*#^	98.45±44.65	*F*_Interaction_ (*P*): 2.438 (0.090)
Value of the gravity of the prefrontal lobe	Before the intervention	60.08±14.57	59.40±12.47	*F*_time_ (*P*): 5.982 (<0.001)
	Two weeks after the intervention	57.91±12.88	54.87±9.92	*F*_group_ (*P*): 3.909 (0.049)
	At the end of the intervention.	55.88±12.30*	51.01±9.62*	*F*_Interaction_ (*P*): 0.821 (0.441)
Bilateral temporal lobe integral value	Before the intervention	84.05±61.16	85.34±61.18	*F*_time_ (*P*): 22.657 (<0.001)
	Two weeks after the intervention	103.16±31.56	116.25±32.49^*^	*F*_group_ (*P*): 4.423 (0.037)
	At the end of the intervention.	124.38±57.97^*^	153.62±39.20^*#^	*F*_Interaction_ (*P*): 1.246 (0.289)
Bilateral temporal lobe barycenter values	Before the intervention	60.14±7.88	60.21±7.69	*F*_time_ (*P*): 10.081 (<0.001)
	Two weeks after the intervention	58.41±6.53	57.13±6.40	*F*_group_ (*P*): 5.573 (0.019)
	At the end of the intervention.	57.78±6.95	53.23±5.49^*#^	*F*_Interaction_ (*P*): 3.118 (0.046)

Compared with before intervention, ^*^P < 0.05; Compared with the same group after two weeks of intervention, ^#^*P* < 0.05.

### Neurotransmitters

**Figure 6:**
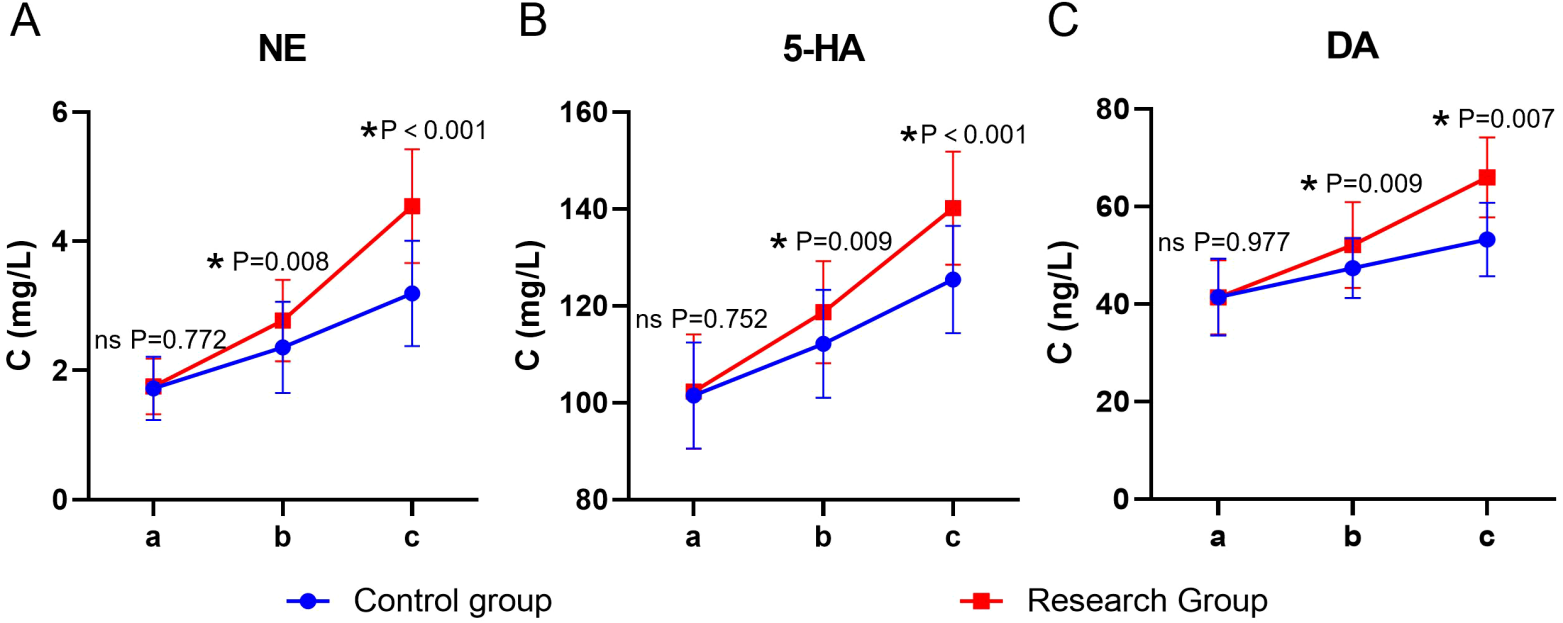
Neurotransmitters in both groups. **(A)** NE, **(B)** 5-HT, **(C)** DA; a: before the intervention, b: two weeks after the intervention, and c: at the end of the intervention. NE, Norepinephrine; 5-HT, serotonin; DA, Dopamine. ns, no significance, *P < 0.05.

Significant main effects of time and group, as well as a significant time × group interaction, were observed for all neurotransmitter levels (*P* < 0.05). The simple effect analysis indicated that before the intervention, there was no statistically significant difference in the levels of NE, 5-HT, and DA between the two groups (*P* > 0.05); at the end of the intervention, the levels of NE, 5-HT, and DA in the study group were higher than those in the control group, and the differences were statistically significant (*P* < 0.05). The intra-group comparison showed that the scores of both groups significantly increased compared to the baseline level after the intervention (*P* < 0.05) (**Table 7**, **Figure 6**).

**Table 7:** Neurotransmitters in both groups (n = 40, mean ± SD).

Group	Group	Control group	Research group	*F* (*P*)
NE	Before the intervention	1.72±0.49	1.75±0.43	*F*_time_ (*P*): 70.829 (<0.001)
	Two weeks after the intervention	2.36±0.71^*^	2.77±0.63^*^	*F*_group_ (*P*): 20.380 (<0.001)
	At the end of the intervention.	3.19±0.82^*#^	4.54±0.88^*#^	*F*_Interaction_ (*P*): 10.424 (<0.001)
5-HT	Before the intervention	101.47±10.97	102.28±11.83	*F*_time_ (*P*): 95.188 (<0.001)
	Two weeks after the intervention	112.18±11.13^*^	118.71±10.53	*F*_group_ (*P*): 13.333 (<0.001)
	At the end of the intervention.	125.44±11.05^*#^	140.15±11.68^*#^	*F*_Interaction_ (*P*): 3.306 (0.038)
DA	Before the intervention	41.40±7.89	41.35±7.61	*F*_time_ (*P*): 92.101 (<0.001)
	Two weeks after the intervention	47.39±6.18^*^	52.11±8.84^*^	*F*_group_ (*P*): 25.838 (<0.001)
	At the end of the intervention.	53.26±7.55^*#^	65.94±8.24^*#^	*F*_Interaction_ (*P*): 10.066 (<0.001

Compared with before intervention, ^*^P < 0.05; Compared with the same group after two weeks of intervention, ^#^*P* < 0.05.

## Discussion

This study, using fNIRS technology, systematically evaluated the clinical efficacy of rTMS in adolescent depression patients and its impact on neurotransmitter levels. Results showed that rTMS significantly improved depression and anxiety symptoms, enhanced quality of life, and effectively regulated neurotransmitter levels. These findings confirmed rTMS’s potential in adolescent depression treatment and provided new insights into its mechanisms.

The core mechanism of rTMS involves generating a time-varying magnetic field that non-invasively penetrates the skull and induces electrical currents in the target brain region. This electromagnetic stimulation can specifically regulate neuronal membrane potentials and alter cortical excitability (13). The study found that the active rTMS group had significantly lower HAMD and HAMA scores compared to the sham-stimulated control group (*P* < 0.05). The induced currents could regulate the discharge patterns of pyramidal neurons in the prefrontal cortex, effectively alleviating the core symptoms of depression and anxiety. Additionally, the rTMS group showed significant improvement in all dimensions of the quality-of-life scale (*P* < 0.05), indicating that magnetic stimulation not only improved mental symptoms but also enhanced social functioning and quality of life by regulating neural network activity. This finding is consistent with the study by Aditya Somani et al. (14), which confirmed that high-frequency rTMS (10 Hz) stimulation of the left DLPFC is very effective in improving depression. In addition to depression symptoms, rTMS also showed significant efficacy in alleviating coexisting anxiety symptoms. A recent meta-analysis by Sai Krishna Tikka et al. (15) revealed similar treatment trends in patients with coexisting OCD and GAD. Overall, these findings suggest that rTMS may improve a range of mental symptoms by regulating the prefrontal - limbic system neural circuitry.

Cognitive function, an integrated ability of brain information processing, depends on the coordinated operation of multiple functional brain regions. In clinical assessments, the DSST, DST, and VFT are typical measures for evaluating cognitive function. The DSST focuses on information processing speed and visuomotor coordination, the DST emphasizes working memory capacity and sustained attention, and the VFT mainly reflects semantic memory retrieval and language organization ability (16). The study found that the rTMS group showed significantly greater improvement in DSST, DST, and VFT scores compared to the control group (*P* < 0.05). This finding can be explained by the following neural mechanisms: First, rTMS may enhance the functional connections between the prefrontal cortex and the temporal lobe through white matter bundles such as the uncinate fasciculus. This structural connection improvement directly promotes the efficiency of semantic information retrieval and language fluency (17). Second, targeting the characteristic hyperactivity of the default - mode network (DMN) in depression patients, rTMS may regulate neural network balance by inhibiting the hyperactivity of the DMN while enhancing the functional efficiency of task - related networks (such as the central executive network) (18). More importantly, the synaptic plasticity changes induced by rTMS have a cumulative effect, and repeated stimulation triggers a continuous process of synaptic remodeling. This feature distinguishes rTMS from drug treatments that can only temporarily regulate neurotransmitter levels (19). In summary, rTMS effectively promotes the multidimensional improvement of cognitive function in depression patients through a variety of neurobiological mechanisms, including regulating prefrontal cortical neural activity, enhancing neural plasticity, and balancing the neurotransmitter system. These neurobiological changes provide a solid theoretical basis for the application of rTMS in depression treatment.

In exploring the neural pathological mechanisms of depression, functional abnormalities in the prefrontal cortex and temporal lobe are key components (20). The prefrontal cortex, a core brain region for emotional regulation, cognitive control, and decision - making, shows functional impairment characterized by reduced metabolic levels, decreased blood flow, and impaired neural plasticity. These changes are closely related to the symptoms of low mood and cognitive decline observed in depression patients (21). At the same time, hippocampal atrophy within the temporal lobe structure is clearly related to memory impairment and abnormal emotional regulation (22). In this study, it was observed that rTMS intervention significantly increased the integral values and decreased the central values of the prefrontal cortex and bilateral temporal lobes (*P* < 0.05). These changes have important neurophysiological significance and reflect the specific regulatory effects of rTMS on brain functional networks. The increase in integral values indicates enhanced overall neural electrical activity in the target brain regions, possibly due to rTMS - induced synaptic plasticity and functional connectivity improvements. This change in neural plasticity helps improve the efficiency of information transmission within neural circuits. The decrease in central values indicates that neural activity is more concentrated in specific sub - regions related to function (such as the DLPFC or the left superior temporal gyrus) (23). This spatial reorganization may optimize brain information processing and lead to more efficient allocation of neural resources. Regarding potential mechanisms, the eddy currents generated by the electromagnetic induction of rTMS can directly regulate the permeability of neuronal ion channels and change local field potential activity (24). In addition, the polarized currents induced by the time - varying magnetic field can specifically regulate the electrical activity of neural tissue. The synergistic effects of these biophysical mechanisms not only enhance the overall neural excitability of the target brain regions but also concentrate neural activity in specific sub - regions related to function, thereby achieving selective regulation of brain functional networks (25). This finding provides important physiological evidence for understanding the neural regulatory mechanisms and therapeutic effects of rTMS.

Neurotransmitter imbalance is widely considered a core pathological mechanism of depression (26). Depression patients often show low levels of NE, especially in key brain regions for emotional regulation such as the prefrontal cortex and amygdala, which can lead to symptoms such as low mood, fatigue, and loss of interest. 5 - HT is a key neurotransmitter that regulates mood, sleep, appetite, and cognitive function. Imbalances in 5 - HT can lead to mood swings and abnormalities in sleep and appetite. DA is closely related to reward, motivation, pleasure, and drive. Its reduction in depression patients can lead to decreased reward perception and exacerbate depressive symptoms (27). In this study, it was observed that after rTMS intervention, the levels of NE, 5 - HT, and DA in the rTMS group were significantly improved compared to the control group (*P* < 0.05). This result can be attributed to the multi - level mechanisms of rTMS. At the neural circuit level, rTMS stimulation of the DLPFC can simultaneously regulate multiple monoaminergic nuclei. Specifically, it enhances 5 - HT neuronal activity through the thalamocortical - midbrain reticular nucleus circuit, promotes NE release through the prefrontal cortex - locus coeruleus pathway, and activates DA neurons through the prefrontal cortex - ventral tegmental area circuit (28). At the molecular level, rTMS improves synaptic plasticity through long - term potentiation (LTP), upregulates brain - derived neurotrophic factor (BDNF) expression, and regulates the activity of monoamine synthetase (29). This multi - target regulation, combined with immediate electrophysiological effects and long - term changes in neural plasticity, constitutes the basis for the comprehensive improvement of clinical symptoms in depression patients.

Although the present study provides important evidence for the efficacy and underlying mechanisms of rTMS in adolescent depression, several limitations should be acknowledged. First, the relatively small sample size may have limited statistical power and the generalizability of the findings; future trials should recruit larger cohorts to enhance the robustness of the conclusions. Second, because all participants were recruited from an outpatient setting, patients with more severe illness or longer disease duration were excluded, potentially introducing selection bias; subsequent studies should enroll patients across a wider spectrum of severity and compare their remission and response rates. Third, the short follow-up period precluded a comprehensive evaluation of the long-term efficacy and safety of rTMS; longer-term follow-up is therefore warranted to determine the durability of the observed benefits. Finally, although fNIRS enables real-time monitoring of cerebral function, its spatial resolution is limited; integrating high-resolution imaging techniques such as fMRI will be essential for more precisely elucidating the neuroregulatory mechanisms of rTMS.

In summary, rTMS regulates neural electrical activity and neurotransmitter release in target brain regions through electromagnetic induction generated by a time - varying magnetic field, thereby alleviating depressive symptoms in adolescents. Future research should further elucidate the dose - response relationship between different magnetic field parameters and therapeutic effects and explore the synergistic effects of magnetic stimulation with other neuro - modulation techniques to develop more personalized treatment plans.

## Data Availability

The raw data supporting the conclusions of this article will be made available by the authors, without undue reservation.
